# The Royal Northern Hospital

**Published:** 1923-08

**Authors:** 


### THE ROYAL NORTHERN HOSPITAL.

### NEW CASUALTY DEPARTMENT.

LADY PATRICIA RAMSAY laid the Foundation Stone of the new Casualty Department of the Royal Northern Hospital, Holloway, on July 14. This new building is the Borough of Islington's War Memorial to over 1,270 Islington men and two women (one a nurse) who fell in the war. There could be no fitter memorial than this provision for the treatment of casualties occurring in the district from which those who paid the supreme sacrifice were taken.

### EXPEDITION AND EFFICIENCY.

The present inconvenient Casualty Department often deals with 120 cases daily. The new building will make for expeditiousness, efficiency and asepsis. The ground floor is united to the existing departments by a wide corridor, with a large waiting hall, enquiry office, two isolation rooms (with external access), nurses' and store rooms, an operating theatre (with a north light) with two recovery rooms and a preparation room adjoining the surgery for casualties, which will contain six examination cubicles. The first floor, to be reached by a staircase and passenger lift, commodious enough for a wheeled stretcher, will consist of nine single bed observation wards, two children's wards, of four beds each, a service room, lavatories and bath rooms.

### THE £50,000 DEFICIT.

Lady Patricia inspected two guards of honour on her arrival, one consisting of ex-Service men, the other of sisters and nurses. The Highgate Silver Prize Band played outside the marquee, and a hymn was beautifully sung by a choir of nurses. A small patient presented a bouquet of carnations to Lady Patricia, who was received by the Mayor of Islington and by the Marquess of Northampton, chairman of the Hospital, who said that it must not be thought the Hospital was pecuniarily flourishing because of the new development. That was solely due to the Islington War Memorial—money contributed to it could not be devoted to reducing the £50,000 deficit. If at least £20,000 were not raised by July 31 70 beds would have to be closed.

